# Move Your Body, Boost Your Brain: The Positive Impact of Physical Activity on Cognition across All Age Groups

**DOI:** 10.3390/biomedicines11061765

**Published:** 2023-06-20

**Authors:** Felice Festa, Silvia Medori, Monica Macrì

**Affiliations:** Department of Innovative Technologies in Medicine & Dentistry, University “G. D’Annunzio” of Chieti-Pescara, 66100 Chieti, Italy

**Keywords:** age groups, attention, brain, cognition, default mode network, executive function, exercise, magnetic resonance imaging, memory, mental health

## Abstract

While the physical improvements from exercise have been well documented over the years, the impact of physical activity on mental health has recently become an object of interest. Physical exercise improves cognition, particularly attention, memory, and executive functions. However, the mechanisms underlying these effects have yet to be fully understood. Consequently, we conducted a narrative literature review concerning the association between acute and chronic physical activity and cognition to provide an overview of exercise-induced benefits during the lifetime of a person. Most previous papers mainly reported exercise-related greater expression of neurotransmitter and neurotrophic factors. Recently, structural and functional magnetic resonance imaging techniques allowed for the detection of increased grey matter volumes for specific brain regions and substantial modifications in the default mode, frontoparietal, and dorsal attention networks following exercise. Here, we highlighted that physical activity induced significant changes in functional brain activation and cognitive performance in every age group and could counteract psychological disorders and neural decline. No particular age group gained better benefits from exercise, and a specific exercise type could generate better cognitive improvements for a selected target subject. Further research should develop appropriate intervention programs concerning age and comorbidity to achieve the most significant cognitive outcomes.

## 1. Introduction

Physical activity (PA) is defined as “any bodily movement produced by skeletal muscles that requires energy expenditure” [1] (p. 126) and can encompass several activities, such as housework, sports, and active recreation [2]. Limitations to or, often, a complete absence of exercise cause various health problems, including postural and somatic disorders, diabetes, overweight, obesity, cardiovascular disease, and even premature death [3,4,5]. Existing literature suggests that regular PA, particularly aerobic activity, promotes physical and mental improvements in healthy and impaired people [6]. 

While several papers have dealt with exercise-induced benefits on body health, only in recent years has the study of the relationship between PA and cognition received considerable attention. This relationship directly influences cognitive functioning associated with structural and functional changes in the brain and improves psychophysical well-being [7]. Although a definition of cognition has yet to be standardized [8], the term cognition includes a series of mental abilities that enable us to perceive, process, and store valuable information in daily life. It has been widely demonstrated that PA can positively influence different cognitive functions (e.g., attention, memory, and executive functions) and has more generic effects on the mechanisms involved in learning. Pioneering research on rodents demonstrated how PA could induce growth factor production and changes in the hippocampus, improving memory [9]. These favourable outcomes on mice have spurred research on humans.

The first human studies indicated that these benefits are mediated by complex neurophysiological and biochemical systems, such as the brain-derived neurotrophic factor (BDNF), which over time, leads to a more efficient, adaptive, and plastic brain [10]. In humans, circulating BDNF and vascular endothelial growth factors are acutely and chronically enhanced after aerobic exercise, leading to brain structure and function changes and neurogenesis [11]. Considering recent mouse studies, the expression of BDNF and cytokines following a systemic administration of myokines released during muscular contraction, such as irisin, could have an anti-depressant effect [12]. However, these neurobiological mechanisms have yet to be understood entirely [13], primarily due to a need for suitable procedures for evaluating brain function in vivo during a dynamic motor task.

Cognitive functioning is commonly assessed through mental status examinations, which evaluate the level of consciousness, orientation, constructional ability, and cognitive abilities. Nowadays, neuroimaging methods detail the link between exercise and cognitive function, provide the physical-exercise-induced changes in the neural correlates of cognition in both short-term and long-term practice, and assess brain health [14]. Among the methods available to investigate the impact on functional brain activation, functional near-infrared spectroscopy (fNIRS), electroencephalography (EEG), and structural and functional magnetic resonance imaging (MRI) have been used in previous research [15,16]. fNIRS and EEG have a limited spatial resolution and may only allow for the evaluation of brain activation patterns in superficial cerebral areas. However, functional magnetic resonance imaging (fMRI) has recently overcome these shortcomings.

fMRI is a fundamental non-invasive instrument of investigation to acquire high-definition images of whole brain activity during different motor tasks and to estimate the brain activation changes in cortical and subcortical areas (cerebral networks) via the blood oxygenation level-dependent (BOLD) signal [17,18,19]. The human brain comprises anatomical regions (nodes or hubs) and synaptic connections among the various regions (edges) [20]. The cerebral networks may change after PA and usually encompasses all body muscles, or after jaw-related therapeutic interventions that involve especially masticatory muscles [21]. Task-based fMRI and resting-state fMRI examine BOLD changes obtained during a cognitive task and while performing no straightforward task, respectively. These two methods are suitable for exploring the modification in neural correlates driving cognitive improvements and measuring brain functional connectivity after exercise or other environmental factors, such as thermal stress [22]. Functional connectivity relates to areas of the brain that are spatially separated but temporally connected in their signalling [23]. A good use of fMRI is in the preclinical models [24]. Indeed, fMRI may even detect abnormal mitochondrial functions, especially in severe inheritable metabolic diseases with neurological manifestations [25].

Previous reviews have investigated the current literature concerning the link between chronic exercise and cognition, mainly among older adults [26,27,28]. These reviews generally encompassed studies evaluating exercise-induced changes at the molecular and cellular levels and in cognitive functions. Moreover, most papers analysed the impact of PA in patients with mild cognitive impairment [29]. Few fMRI reviews dealt with older people and reported better functional connectivity, especially after light-intensity aerobic exercise [30,31]. Recent studies pointed out the positive impact of exercise since PA could counteract the loss of brain white and grey matter and promote the efficiency of brain circuits and neuronal plasticity, especially in adults [32,33]. Regarding children, most papers evaluated the impact of PA through standardised tests of academic and cognitive outcomes. Few reviews included neuroimaging effects following exercise [34,35]. A 2019 review examined cognitive improvements among elders, adults, and children through neuropsychological tests but had only two fMRI studies concerning children [36]. Therefore, the authors did not provide a complete analysis of functional changes during the lifetime of a person.

To provide a more up-to-date and in-depth analysis, we described the relationship between PA and brain health using standardised tests, and structural and functional MRI in this review. **We reviewed recent, peer-reviewed studies with original data and published in English that concerned the effects of exercise and the cognitive benefits across all age groups. We investigated the effect of acute and chronic PA. Acute PA indicates a single bout of physical exercise, while chronic PA indicates PA that is repeated and persists longer than a single session or episode. Acute PA studies showed the transitory response to a single session of PA, whereas chronic PA illustrated a long shift in an individual’s life. Due to heterogeneity among the studies, the studies selected were divided into “acute exercise” and “chronic exercise” to provide readily comprehensible data.** We used PA or exercise to refer to coordinative activities (e.g., yoga, Tai Chi exercise, and dance) and aerobic fitness (e.g., walking, cycling, and treadmill running).

The present narrative review aimed to understand what mechanisms and brain changes were involved following acute and chronic PA in older people, adults, and children; whether a specific age showed the most significant cognitive improvements following PA; and lastly, whether a particular exercise type could provide better cognitive performances in a selected age group.

## 2. Acute Exercise

Previous studies have targeted cognitive abilities as evidence suggests that a single exercise session may temporarily alter them [37]. A 2001 study observed that older patients with chronic obstructive pulmonary disease (COPD) showed better verbal fluency following 35 min of acute aerobic exercise (20 min cycling and 15 min recovery period) [38]. **Therefore, acute exercise positively impacts both healthy and impaired people.** Although previous papers recommended acute physical exercise [39], the relationship between acute PA and cognition remains to be determined. **Consequently, we investigated the cognitive improvements** in subjects of different ages via fMRI and the cognitive test for attention, cognitive control, or memory, especially following a single cycling or treadmill running session (Table 1).

Regarding older adults, a low level of PA is considered a risk factor for dementia. Acute aerobic exercise increased the BDNF plasma levels in patients with Alzheimer’s disease and healthy controls. In addition, the BDNF levels had an association with the level of PA [40]. Following 30 min of moderate-intensity exercise in healthy older adults, higher activation was observed in the middle frontal, inferior temporal, middle temporal, and fusiform gyri during a semantic memory task and in the bilateral hippocampus after exercise compared with at rest [41]. Moreover, 30 min moderate-intensity bicycling generated greater activation in the left inferior frontal gyrus and left inferior parietal lobule and deactivation in the right anterior cingulate gyrus [42]. Functional connectivity did not differ significantly between acute light- and moderate-intensity training in cognitively normal older adults. A correlation between the right postcentral/parietal cortex, the right ventral lateral prefrontal cortex, the right posterior superior temporal gyrus, and the right dorsolateral prefrontal cortex was detected after acute exercise [43]. In older adults, 20 min cycling, especially at moderate intensity, improved working memory [43], while 30 min moderate-intensity cycling enhanced executive function and functional processing [42].

Regarding younger adults, we reported some studies concerning the influence of different duration and intensities of PA. Recent papers have reported that a single session of PA increased functional connectivity in specific brain networks. These findings are essential for cognitive and motor functions, often the primary goal of neurorehabilitation strategies [56]. Suwabe et al. observed that young adults showed a higher hippocampal memory function thanks to better functional connectivity between dentate gyrus and cortical networks following a 10 min, very light-intensity PA bout [44]. According to Li et al., in younger adults, a 20 min moderate-intensity physical session slightly improved working memory; however, this single bout generated a greater activation in the right middle prefrontal gyrus, the right lingual gyrus, and the left fusiform gyrus [45]. The improvement in executive control processes after acute exercise was related to activation of the prefrontal and occipital cortexes and deactivation of the anterior cingulate cortexes and left frontal hemisphere. Moreover, motor sequence memory in connection with the hippocampus increased significantly for high-intensity PA while tending towards significance for moderate PA in healthy young males; the bilateral precuneus activity improved for both moderate- and high-intensity PA [46]. The mnemonic discrimination task, working memory, and executive function were enhanced in younger adults after 20 min moderate-intensity cycling, while executive function improved in younger adults with attention-deficit/hyperactivity disorder (ADHD) after 30 min moderate-intensity cycling. A single bout of aerobic exercise significantly improved learning mechanisms in young male adults’ visual and motor domains. These benefits could last for at least 30 min after exercise. Moreover, moderate-intensity acute PA could allow for a gradual up-regulation of a functional network due to a constant rise in synapse strength, which could encourage brain plasticity in motor and non-motor areas [47]. Mehren et al. analysed the impact of a 30 min single session of aerobic exercise on attention and executive functions in adult patients with and without ADHD. Moderate-intensity PA significantly improved reaction times in patients with ADHD compared with healthy adults [48]. Although the authors noticed no changes in brain activation between the two groups, they supported the importance of acute exercise for patients with ADHD. In another study, Mehren et al. compared moderate-intensity exercise with high-intensity exercise in healthy younger adults [49]. A better behavioural performance (sensitivity index) and greater activation in areas related to executive function, attention, and motor processes (insula, superior frontal gyrus, precentral gyrus, and supplementary motor area) was noticed following moderate PA. Higher cardiorespiratory fitness was also linked to increased brain activation of the right insult and left rolandic operculum after moderate exercise and decreased brain activation of the right postcentral gyrus after high-intensity exercise. Thirty minutes of low-intensity acute exercise in healthy male athletes led to reduced brain activation in the posterior cingulate cortex/precuneus. In contrast, a 30 min high-intensity acute workout reduced the caudate nucleus and ventral anterior putamen [50]. Schmitt et al. also described a positive interference of intense acute PA in emotion-processing brain regions during fearful face elaboration [50]. The authors concluded that single acute exercise sessions are usually beneficial for mood. Moreover, after 30 min acute aerobic exercise, Li et al. noticed that the right cerebellum played a decisive role in processing simple executive tasks [51]. At the same time, the subcortical regions were involved in the processing of relatively complex executive tasks.

A few papers dealt with acute PA’s impact on adolescents and children. Acute aerobic stretching and moderate-intensity exercise affected the theta and alpha waves of the EEG and, thus, had beneficial effects on brain maturation and development of children aged 12 to 14 years, especially those with ADHD [52]. Executive function and working memory performance improved in healthy children after 30 min moderate-intensity cycling [53,54]. During a working memory task, a more significant change in functional brain haemodynamics was also detected in the bilateral parietal cortexes, the left hippocampus, and the bilateral cerebellum. A 30 min cycling session also led to higher brain connectivity between the right dorsolateral prefrontal and left cerebellum, inversely associated with improved cognitive performance. Metcalfe et al. observed no significant modifications in the attentional task after 20 min moderate-intensity cycling in adolescents with and without bipolar disorder [55]. In adolescents with bipolar problems, the authors noticed a reduced functional activation in the orbital part of the left inferior frontal gyrus, the right frontal pole extending to the temporal pole, the bilateral hippocampus, and the right amygdala.

Regardless of age, brain changes following acute exercise were mainly found in the frontal and temporal lobes, the cerebellum, and the hippocampal regions. Moreover, executive functions associated with the frontal lobe and hippocampus could be selectively maintained or enhanced in humans with higher fitness levels. Herold et al. underlined that different acute exercise protocols (cycling or treadmill running) and various intensities (light, moderate, and high), as well as the cardiorespiratory fitness level and sex of the participants, affect PA-related shifts in functional brain haemodynamics [57].

## 3. Chronic Exercise

**Figure 1:**
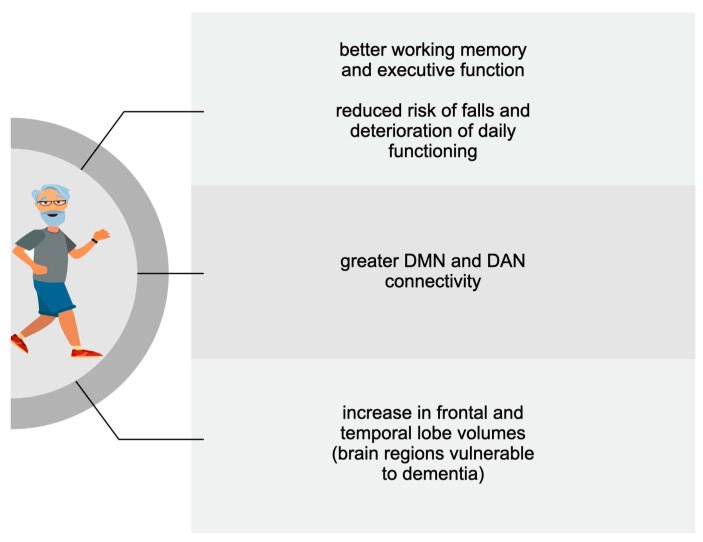
The main cognitive effects of chronic exercise among older adults.

**Figure 2:**
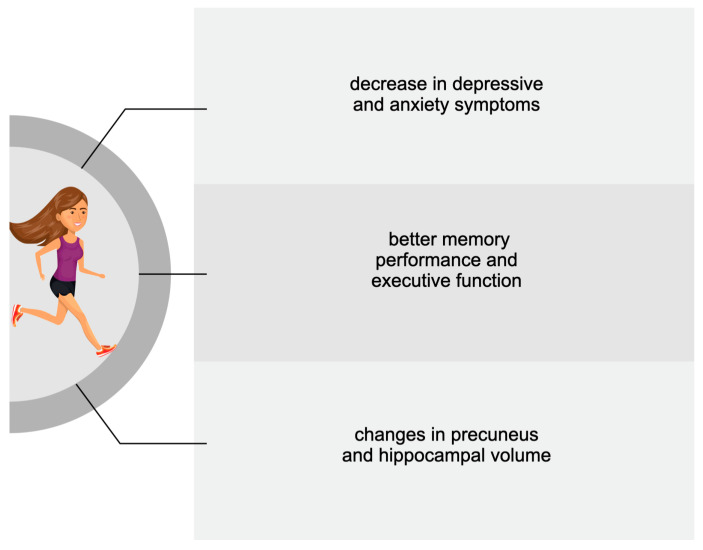
The main cognitive effects of chronic exercise among adults.

**Figure 3:**
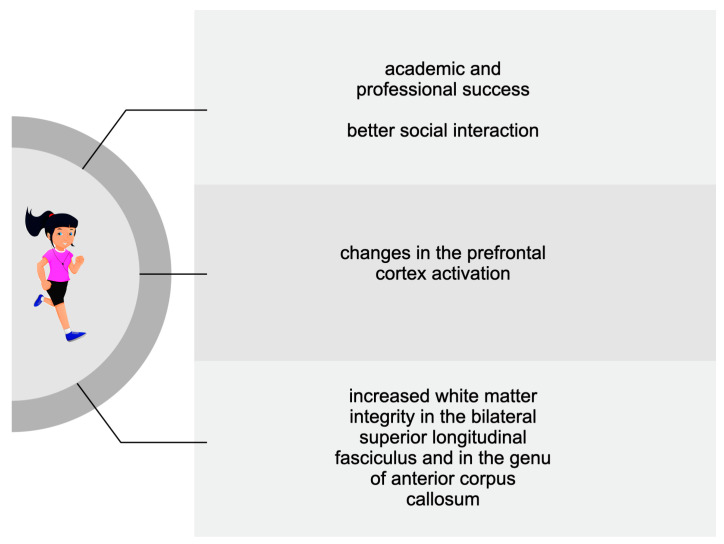
The main cognitive effects of chronic exercise among adolescents and children.

A more significant number of studies dealt with the benefits of PA on cognition following a period longer than a single bout. Therefore, descriptions of the positive impact of regular exercise have been divided according to the age of the subjects analysed in the reviewed studies. **Figure 1, Figure 2 and Figure 3 summarise the main PA-induced effects on cognition among older adults, adults, adolescents, and children. These benefits are described in detail in the following subsections.**

### 3.1. Older Adults

The elderly population is increasing worldwide; according to the World Health Organization (WHO), by 2030, one in six people will be 60 years or over [58]. This leads to an increasing interest on the part of the scientific research community in ageing and the quality of life of the elderly population. With age, cognitive impairments, such as dementia and Alzheimer’s disease, may occur in addition to physical conditions. Therefore, older people should implement preventive strategies and lead healthy lifestyles. Several risk factors, for instance, smoking, physical inactivity, being overweight, and high blood pressure, may contribute to the onset of dementia and related diseases. Most people need to be fully aware of the relationship between lifestyle and brain health, especially in subgroups with low socioeconomic statuses or low levels of social health literacy. It, therefore, appears essential to raise awareness in the general population on modifiable risk and protective factors for dementia, as was the goal in an awareness campaign in the Netherlands, “my brain coach” [59]. Numerous studies pointed out a compatible correlation between regular physical exercise and a lower incidence of dementia and cognitive impairment [60,61,62].

However, the underlying mechanism by which PA may improve cognition is still unknown. Some studies found that the level of training intensity influenced the increase in cognitive ability in older adults [63,64]. In contrast, others stated that simple daily movement was significantly linked to cognition [65,66]. An improved cognition reduces the risks of falls, cognitive complaints, and deterioration of everyday functioning. The duration of PA sessions varied from 60 to 90 min. On the contrary, Langhammer et al. proposed an activity time of 150 min weekly for at least six months [67]. Taylor et al. recommended 30 daily aerobic exercises five days a week and two days of strength-based training [68]. In addition, a systematic review suggested that moderate-intensity exercise programs with aerobic and resistance training, lasting at least 45 min per session on as many days of the week as possible, could benefit older healthy adults’ cognition [62]. Pietrelli et al. analysed the benefits of moderate-intensity aerobic exercise, performed regularly throughout life, on brain health and anxiety-related behaviour in old rats [69]. By studying cognitive response with the radial maze (RM) and anxiety-related behaviours with the open field (OF) and higher maze (EPM), the authors found improved cognitive function and protection from the deleterious effects of ageing and decreased anxiety. Thus, regular and chronic aerobic exercise generated time- and dose-dependent, neuroprotective, and reparative effects on physiological brain ageing, reducing anxiety-related behaviours. With regard to PA type, Klimova et al. reported that dance improved cognitive performance in healthy older people thanks to emotional involvement, balance control, memory, and coordination [70]. On the contrary, the effects of high-intensity interval training (HIIT) interventions on functional brain changes in the elderly remain unclear. A Korean preliminary study demonstrated that a HIIT program that included flexibility, endurance, and balance effectively improved cognitive function, physical fitness, and electroencephalographic markers in older Koreans; thus, HIIT interventions could help improve functional brain activity in this population [71]. Several studies described that exercise could activate different mechanisms at the brain level, promoting various physiologic phenomena, such as angiogenesis, neurogenesis, synaptogenesis, and stimulation of neurotrophic factors improving memory and brain plasticity. PA modulated Aβ turnover, inflammation, the synthesis and release of neurotrophins, and cerebral blood flow in older populations. Moreover, encouraging lifestyle changes in the pre-symptomatic and pre-dementia disease stages could potentially delay a third of dementia worldwide [72].

Table 2 summarises the findings of the following reviewed studies.

Engeroff et al. described the associations of objectively assessed habitual PA and physical performance with brain plasticity in cognitively healthy older adults [73]. The brain plasticity was analysed using magnetic resonance spectroscopy (MRS)-based markers and BDNF serum levels. Overall, PA and exceeding current recommendations for moderate-to-vigorous PA were positively related to BDNF. On the contrary, sedentary behaviour was negatively correlated with the bioavailability of neurotrophic factors in the elderly. Moderate-intensity morning exercise improved serum BDNF and working memory or executive function in older adults, depending on whether the next session was interrupted with light-intensity intermittent walking [74]. A recent study evaluated systemic biomarkers in learning and memory in 23 asymptomatic late middle-aged adults with familiar and genetic AD risk after 26 weeks of supervised treadmill training [75]. Systemic biomarkers included myokine cathepsin B (CTSB), BDNF, and Klotho. The authors observed a modification in the metabolic regulation of exercise-induced plasma BDNF. They concluded that CTSB is a marker of cognitive changes in late middle-aged adults with a risk for dementia.

A study proposed another aspect: the evaluation of older people’s neural activation via electroencephalography (EEG) in response to the environment in which walking was carried out [76]. Levels of ‘engagement’ (related to immersion) were higher in urban green spaces than in crowded or quiet residential areas, while levels of ‘excitement’ (linked to classic arousal indicators such as increased heart rate and blood flow) were higher in busy urban streets than in green areas and quiet urban spaces. Therefore, green spaces in an urban setting could favour walking in older adults.

Some studies aimed to evaluate functional connectivity after PA in older adults. The default mode network (DMN) and the dorsal attention network (DAN) represent the most susceptible brain networks to ageing. The DMN is positioned in the ventromedial prefrontal and posterior cingulate cortex and is active at rest [96]. At the same time, the DAN is located in the intraparietal sulcus and frontal eye fields and is active during tasks that require voluntary and sustained attention [97]. The posterior cingulate cortex controls memory functioning, whereas the left frontal eye field controls attention.

In older adults without significant neurological (e.g., Alzheimer’s and Parkinson’s) or psychiatric diseases, the DMN and DAN were strongly positively correlated with PA. The DMN plays a crucial role in high-level cognitive and self-referential processes; abnormal functioning of the DMN is related to psychological diseases, such as depression, anxiety, and attention deficit. The DAN is involved in top-down control of attention and sensory–motor information integration. The relation between PA and DMN varied depending on the levels of executive function: this association was only significant for high executive function. Moreover, PA was not significantly related to whole brain global or local efficiency [77]. In elders with the lowest DMN/DAN anti-correlation levels, higher PA and fitness were associated with improved executive function [78]. As reported in other studies, better executive function performance would be related to greater DMN and DAN connectivity.

Walking or dancing together with sensorial stimuli (musical stimulation) and fun-recreational activities (for example, book reading, crossword, and sudoku) led to significant changes in DMN, especially the precuneus, the right angular gyrus, and the posterior cingulate cortex, and in DAN, particularly the left frontal eye field [79]. The prefrontal cortex and medial temporal lobe are susceptible to ageing consequences. These areas showed resting-state functional connectivity following Tai Chi exercise, mainly if associated with cognitive training and group counselling [80]. Regarding memory processes, the hippocampus and medial prefrontal cortex communication play a key role [98,99]. Resting-state functional magnetic resonance imaging showed increased functional connectivity between the hippocampus and medial prefrontal cortex after Tai Chi Chuan and Baduanjin practices [81]. Moreover, frontal and subcortical networks also improved after exercise [82].

As concerns duration of PA, 6 weeks of PA produced significant changes in the right striatum (including both the putamen and the globus pallidus) and the posterior cingulate cortex/precuneus area, and no volume reduction in the right striatum [83]. On the contrary, an improvement in functional connectivity between the frontal, posterior, and temporal cortexes within the DMN and the frontal executive network was only found after one year of walking. Voss et al. noticed a non-significant but trending effect on the connectivity of the DMN following 6 months of training [84], which was confirmed by Flodin et al., who reported modulation of mid-temporal brain regions and hippocampus [85]. Voss et al. also suggested a boost in exercise-induced effects on functional connectivity thanks to nutritional supplements, including beta-alanine [86].

As for the intensity of daily PA, easy-paced walking (light activity) generated a greater BOLD in the left prefrontal and parietal regions. At the same time, brisk walking (moderate exercise) induced a greater BOLD response in the dorsolateral prefrontal cortex. Therefore, Kimura et al. concluded that moderate activity could counteract neurocognitive degradations [87].

PA has been positively associated with grey matter integrity. In structural MRI papers, the frontal and temporal lobes, including the hippocampus, were the areas with the most significant benefits from exercise. Higher levels of PA produced an augmentation and preservation of grey matter in the frontal cortex. An increase in medial temporal lobe volume was described in fit older adults [88]; moreover, this effect could be desirable in subjects with a genetic risk of Alzheimer’s disease [100]. Thus, PA-induced modifications seem to limit the ageing effect of temporal lobes. The pro-inflammatory cytokines affect brain ageing negatively and have been linked to dementia. Indeed, Papenberg et al. reported that grey matter volume could be preserved thanks to an active lifestyle [89]. On the contrary, inflammation related to inactivity could lead to a cognitive decline across 6 years.

In cognitively impaired older people, neural regions associated with language, superior-parietal regions, and frontoparietal regions are the most common brain networks involved after exercise. In older adults with mild cognitive impairment, improvements in functional connectivity between the posterior cingulate cortex and bilateral medial prefrontal cortex and between the posterior cingulate cortex and left hippocampus, as well as a trend of reduced bilateral hippocampal volume atrophy, were noticed after 8 weeks of mindfulness (non-judgmental moment-to-moment awareness obtained through sitting and walking meditation, body scan, and yoga) [90]. The elders who underwent a 12-week yoga intervention showed an enhancement of memory functions and functional connectivity related to verbal, attentional, and self-regulatory performance [91]. Yoga also improved connectivity between the DMN and lingual network, which is positively related to better memory performance. Moreover, Baduanjin training increased grey matter volume in the hippocampus, functional connectivity between the hippocampus and angular gyrus, and the amplitude of low-frequency fluctuations in the anterior cingulate cortex, compared with brisk walking and non-exercise. Tao et al. suggested the potential of Baduanjin in preventing the progression of mild cognitive impairment [92]. Progressive resistance training decreased posterior cingulate–anterior cingulate cortex functional connectivity. Suo et al. also described a greater cortical thickness in the posterior cingulate after 6 months of progressive resistance training [93]. The authors concluded that this mechanism might benefit long-term protection against further cognitive decline, as posterior cingulate grey matter loss is a biomarker of Alzheimer’ s disease [101].

Stroke survivors frequently show cognitive impairments; hence, a selected PA intervention could improve post-stroke recovery. Aerobic exercise provides cognitive benefits, enhancing memory and attention after stroke [102]. The positive exercise-induced effects on cognition are often present even in the chronic stroke phase [103]. Moreover, combined interventions substantially impacted cognition improvement, especially regarding executive function [104]. However, the mechanisms underpinning these positive effects have yet to be fully understood. The following studies described functional brain changes following PA. Hsu et al. investigated the effect of 6 months of progressive aerobic exercise training in subjects with mild subcortical ischemic vascular cognitive impairment [94]. Six months of walking outdoors with progressive intensity did not significantly increase frontoparietal network (FPN) connectivity; that, however, was especially linked to improved mobility performance. Older people stroke survivors showed a positive correlation between daily exercise and DAN [95]. Stroke patients also improved attention performance thanks to PA.

### 3.2. Adults

The studies concerning the impact of chronic PA in adults encompassed a wide range of ages, from young adults to middle-aged people (Table 3). They mainly described the cognitive improvement to counteract psychological disorders and brain ageing. Moreover, the papers concerning adults embraced patients with psychological disorders and healthy people.

Goldin et al. investigated the effects of mindfulness meditation associated with Hatha yoga and 8 weeks of aerobic exercise in patients with social anxiety disorder [105]. Both mindfulness and aerobic training significantly reduced social symptoms and increased mindfulness skills. Nevertheless, mindfulness meditation generated excellent functional connectivity in the posterior cingulate and dorsomedial prefrontal cortex and improved attention-related parietal cortical regions [106]. Eight weeks of exercise in adults with major depressive disorder led to a marginal reduction in hippocampal activations; therefore, exercise reduced the level of depressive disorder from severe to mild [107]. Huang et al. investigated the changes in brain activity in adults aged between 18 and 50 years with and without subthreshold depression/subsyndromal depression before and after 8 weeks of moderate-intensity aerobic exercise [108]. All individuals showed changes in the right precuneus, right fusiform gyrus, right middle cingulate, and right superior parietal lobule and an augmentation of precuneal activity. The right inferior parietal lobule activity was enhanced only in subjects with subthreshold depression. The authors reported a decrease in depressive and anxiety symptoms and underlined the importance of PA in preventing the development of major depression.

Regarding healthy adults, Stern et al. compared the effects of 6 months of aerobic PA with that of stretching/toning exercises on the cognition and brain structure of healthy adults aged 20 to 67 years [109]. Executive function improved significantly after aerobic PA and increased with the subject’s age. Increased cortical thickness in the left caudal middle frontal cortex Brodmann area was observed after aerobic PA. Another recent MRI study reported significantly increased cortical thickness following 6 months of aerobic and anaerobic exercise [110].

Kaiser et al. evaluated the changes in hippocampal volume, vasculature, neuro metabolites, and peripheral growth factors after a 12-week low- (toning) or high-intensity (aerobic) exercise program [111]. The concentrations of N-acetyl aspartate in the dorsal anterior cingulate cortex and BDNF, and peripheral insulin-like growth factor-1 (IGF-1), as markers for neuronal development, were positively linked to cardiorespiratory fitness changes. Moreover, the authors found an improvement in cardiorespiratory function, an increase in left hippocampal, and a decrease in right hippocampal volume, particularly after the high-intensity exercise condition.

Fontes et al. detected the associations of the precentral gyrus and cerebellar vermis with dynamic cycling activity using an fMRI-compatible cycling ergometer [112]. The authors found that the posterior cingulate cortex and precuneus were linked with higher levels of perceived exertion in healthy male young adults. Hence, the primary motor cortex and cerebellum regulate motor activity during cycling. In another study, Ishihara et al. described the cognitive impact of exercise in a broad sample of healthy young adults aged 22–37 years, focusing on the concomitant and independent relations between PA and working memory domains [113]. Superior working memory was linked to higher cardiorespiratory fitness and hand dexterity, mediated by task-evoked functional activity in parts of the FPN and DMN; instead, gait speed and muscular strength did not affect working memory.

As concerns exercise intensity, compared with low-to-moderate-intensity PA (for instance, walking), regular moderate-to-vigorous-intensity PA was associated with better cognitive and mental health measures in young people aged between 20 and 39 years [114].

Regarding healthy middle-aged adults, light- or moderate-intensity PA could lead to functional neuroplastic changes. After short-duration training, Bezzola et al. noticed a significant reduction in neuronal recruitment in the right and left dorsal premotor cortex [115]. Seven days of PA led to a bilateral cerebellar activation. Moreover, better sequence-specific temporal performance generated greater activation in the precentral gyrus, middle occipital gyrus, and putamen of the right hemisphere and the thalamus, cuneus, and cerebellum of the left hemisphere, which was linked to speed rather than the precision of movements [116]. Six months of moderate exercise in sedentary males produced brain activity in bilateral frontal regions that depended on individual fitness gains [117].

As reported above, the studies concerning cognitively normal adults mainly focused on the link between PA, and memory performance and executive function since both cognitive domains are affected by ageing and may change according to age.

### 3.3. Adolescents and Children

Several papers emphasised the importance of chronic PA during childhood and adolescence to prevent cardiac, muscular, and metabolic diseases and to improve cognitive function and scholastic performance [118]. A previous meta-analysis evaluated the correlation between regular physical exercise lasting at least one month and children’s executive functions and noticed a small and measurable improvement in neuropsychological tests of executive functions, mainly inhibitory control. Some studies examined whether specific biomarkers (e.g., BDNF, cathepsin B (CTSB), and fibroblast growth factor 21) were involved in brain health after PA. According to Rodriguez-Ayllon et al., no biomarkers mediated the effects of exercise on brain health [119]. On the contrary, a systematic review reported that adolescent athletes show lower serum but higher plasma BDNF concentrations than sedentary individuals [120]. Moreover, exercise could increase serum BDNF concentrations in inactive adolescents to a small extent.

In children, a sedentary life may negatively affect the development of the growing brain. Therefore, promoting PA and reducing sedentary behaviour may preserve mental health in children and adolescents. PA is a simple and important method for improving children’s mental functioning, such as executive function, which is critical to cognitive development [121]. Regular PA may contribute to academic and professional success, as well as success in social interaction [122].

Most of the following reviewed studies evaluated PA’s impact on cognition by neuroimaging (Table 4).

A Swedish study investigated the effects of moderate-to-vigorous PA and screen time on mental health in 1139 Swedish adolescents (mean age 13.4 years). After seven consecutive days, moderate-to-vigorous PA was associated with better mental health, while the opposite was observed following screen time [123]. These associations were inconsistent across time domains, genders, and mental health outcomes. Kamijo et al. detected increased cardiorespiratory fitness associated with improved cognitive control of working memory in preadolescent children following 9 months of randomised control PA compared with that in a waitlist control group [124]. Quinzi et al. analysed the effects of different disciplines on electrophysiological levels and behaviours in children [125]. The racket players showed the most consistent response time and greater attentional control, and the climbers were characterised by more intense top-down anticipatory attention. The martial arts practitioners had the fastest response time and a more speed-oriented response. Nine months of PA in preadolescent children determined changes in physical fitness (maximum oxygen consumption) and brain electrical activity and, significantly, an enhancement in executive control; therefore, exercise could improve infant cognition and brain health [126].

MRI and fMRI papers mainly analysed the impact of moderate-to-vigorous PA on cognition. After 9 months of moderate-to-vigorous aerobic PA, 8- to 9-year-old children showed a reduction in brain activation in the right anterior prefrontal cortex, which was consistent with patterns of young adults [127]. Following exercise, no changes were detected in the anterior cingulate cortex, insula, and occipital cortex. Moreover, white matter microstructure in the genu of the anterior corpus callosum was enhanced: in 7- to 9-year-old children, the fractional anisotropy increased and the radial diffusivity decreased, whereas no changes in axonal fibre diameter were found following 9 months of aerobic exercise [128]. In addition, the children not subjected to aerobic PA showed typical development without any modification of white matter microstructure. The anterior prefrontal cortex and the corpus callosum were involved in cognitive control. A specific corpus callosum development could prevent cognitive and behaviour deficits, e.g., attention-deficit hyperactivity disorder, autism, and schizophrenia.

A more significant number of studies have been carried out on the impact of PA in overweight children. Youth obesity is rising; according to the WHO European Regional Obesity Report 2022, approximately one-third of European children are overweight or obese [135]. A Mexican study reported a reduction in the hippocampal volume and a lower executive cognitive performance on neuropsychological evaluations in overweight/obese 6- to 8-year-old children, positively correlated with the increase in body mass index (BMI) [136]. Therefore, regular PA could reduce BMI and benefit cognitive performance, as described in the following reviewed papers (Table 4).

Nine months of PA led to many cognitive and brain health benefits in both standard and overweight children [129]. Obese children showed a reduction in neuroleptic indices compared with normal-weight children. In addition, normal-weight children exhibited a decrease in visceral adipose tissue associated with faster task performance. A recent study investigated the positive effects of PA on intelligence, executive function, academic performance, and brain outcomes in overweight or obese children [130]. Brain health indicators, including intelligence, executive function (cognitive flexibility, inhibition, and working memory), and academic performance, were assessed using standardised tests, whereas MRI measured hippocampal volume. In total, 109 obese or overweight participants, aged between 8 and 11 years, underwent 90 min of exercise three times a week. Training positively affects intelligence and cognitive flexibility during development among children with overweight or obesity. However, structural and functional brain changes were not identified. Overweight Black and White 7- to 11-year-old children showed improved bilateral prefrontal cortex activity and reduced bilateral posterior parietal cortex activity following 3 months of regular aerobic exercise [121]. As for executive function, an enhancement in mathematics was detected. No dose–response correlation between PA and cognition, and no changes in motor regions (frontal and supplementary eye fields) were observed. The outcomes concerning prefrontal and parietal cortexes are consistent with those observed in older people. The activation of several regions supporting antisaccade performance, i.e., bilateral precentral gyrus, medial frontal gyrus, paracentral lobule, postcentral gyrus, superior parietal lobule, inferior parietal lobule, anterior cingulate cortex, right inferior frontal gyrus, insula, and left precuneus, decreased in overweight, predominantly Black 8- to 11-year-old children after 8 months of aerobic exercise [131]. In contrast, the activation of other areas supporting flanker performance, i.e., left medial frontal gyrus, superior frontal gyrus, middle frontal gyrus, superior temporal gyrus, cingulate gyrus, and insula, was enhanced. Hence, differences in task strategies could modify different neural circuitries in children with higher BMI. Still, whether the functional connectivity changes depended on increased fitness or decreased body fat was determined. Eight months of aerobic exercise was also associated with reduced synchrony with three resting state networks (motor, default mode, and cognitive control networks) and increased synchrony only between the motor network and frontal regions (right medial frontal, middle frontal, and superior frontal gyri) [132]. A decrease in synchrony becomes more focal and refined synchrony in those regions.

The following papers described the effects of PA on white matter integrity in overweight young people via diffusion tensor MRI. Overweight, predominantly Black 8- to 11-year-old children showed changes in white matter integrity after approximately 8 months of aerobic exercise [133]. PA increased white matter integrity in the bilateral superior longitudinal fasciculus due to increased fractional anisotropy and reduced radial diffusivity. The enhancement of white matter integrity in the right superior longitudinal fasciculus indicated greater selective attention. In contrast, the improvement in white matter integrity in the left superior longitudinal fasciculus showed higher teacher ratings of classroom behaviour [133]. In addition, 8 months of PA generated significantly positive change in bilateral uncinate fasciculus, a white matter fibre tract connecting frontal and temporal lobes [134]. These outcomes indicated an enhancement of white matter structural coherence and myelination.

## 4. Discussion

The current review focused on the association between exercise and cognition in every age group. We found a positive correlation between PA and cognition in humans, which was widely supported by MRI and fMRI studies. **Indeed, PA generated substantial changes in functional brain activation and cognitive performance across all age groups. No particular age group showed more advantage from PA. Still, a specific exercise type could improve cognition in selected subjects.**

The papers reviewed here mainly investigated the effect of chronic PA rather than acute PA. Studies concerning acute PA differed in intensity and duration of exercise, whereas they were similar regarding the type of exercise, i.e., treadmill running or stationary cycling. However, acute aerobic exercise would provide more favourable cognitive improvements. It could create a healthy environment by facilitating cortical activity, haemodynamics, and metabolism, especially at moderate intensity [137].

**In line with previous studies** [36,138], **we noted the exercise-induced mechanisms on cognition at multiple levels of analysis in every age group**. Regarding molecular and cellular levels, PA induces the expression of neurotransmitter and neurotrophic factors involved in changes in brain structure and neurogenesis [139]. Indeed, BDNF underpins neuron growth, survival, synaptic plasticity, axonal pruning, and regeneration. Decreased BDNF levels represent a lack of trophic support and may contribute to cognitive impairment in Alzheimer’s disease. In children and older adults, moderate-intensity resistance training was more effective in maintaining or increasing BDNF levels than other exercise types [140]. **Moreover, BDNF seems to mediate the link between exercise and functional connectivity** [141].

Regarding structural and functional brain changes, MRI studies reported increased grey matter volume in brain regions, especially in the hippocampus, prefrontal regions, and caudate nucleus. Exercise is crucial in preventing brain volume loss, a phenomenon linked to brain ageing. Consistent with previous papers [142], we found a positive impact of PA on frontal and temporal lobes; in particular, exercise may reduce the risk of temporal lobe atrophy [143]. PA was also associated with greater white matter integrity. The increase in white matter integrity in bilateral superior longitudinal fasciculus found in children was linked to a reduction in radial diffusivity, in accordance with studies concerning adults [144]. The frontotemporal white matter integrity increased in unfit children and older adults [145], although the extent of integrity could vary in the age group.

Regarding functional brain connectivity, through fMRI, children, adults, and older adults showed significant changes in brain functional connectivity, especially in the frontal lobe, the cerebellum, and the hippocampus after acute PA. These outcomes are consistent with those detected by other functional neuroimaging techniques (i.e., fNIRS) [15]. In older adults, PA led to more excellent DMN connectivity: in older adults without major neurological diseases, the association between PA and DMN was higher for higher executive function and more extended training periods. Moreover, PA generated changes, especially in frontal, temporal, and parietal regions, i.e., areas sensitive to neurodegeneration [100]. FPN and DAN modifications usually occur in young adults and older people. In adults, the changes were detected in the left parietal regions, especially in the precuneus. The precuneus manages highly integrated mental processes and is often involved in the early stages of Alzheimer’s disease and mild cognitive impairments [146]. PA seems to counteract cognitive decline by improving functional integration of the frontoparietal control network via effects on the precuneus [147,148]. PA increased prefrontal cortex activity and reduced posterior parietal cortex activity in children. There was also better functional connectivity in DMN. **Different study designs can explain these results.**

PA improved attention, working memory, and executive function in all ages. Exercise improved performance on various cognitive task categories, including attention, visuospatial functions, information processing, memory, and executive function. Moreover, the benefits of acute exercise could help prepare for situations demanding high executive control (e.g., complex daily tasks or necessary examinations in educational settings) [149]. Increased functional connectivity in DMN and DAN was associated with more significant enhancement in executive function. An improvement in working memory was noticed in several studies after chronic PA [150]: working memory is involved in daily activities, such as performing mathematical calculations, recognising to-do lists, and turning instructions into action plans. Longer PA interventions in young people improved mental health and cognition outcomes, especially neurobiological alterations [151,152]. However, **most papers focused on working memory and executive function, neglecting other cognitive domains.**

When we analysed whether a particular age or population gained the most significant advantages from PA, we noticed no big differences among different ages. Since most studies dealt with older adults and children, available data are insufficient to state which age exhibited the greatest cognitive improvements from PA. However, overweight children and subjects with subthreshold depression or subsyndromal depression seemed to benefit from exercise, which could prevent worsening clinical status. Consequently, future research should investigate whether exercise-induced benefits are more significant in a specific age or population than others.

Lastly, we evaluated whether a selected age or population achieved better cognitive performance for a specific exercise type. The studies focusing on older adults with cognitive impairment and adults with depression comprised participants who underwent light or moderate PA. These subjects were generally involved in coordinated exercise, for instance, yoga, which has beneficial effects on mood, cognitive function, and neural structure and function via lowering stress, reducing inflammation, and improving neuroplasticity processes. Quinzi et al. demonstrated how different sports (racket sport, martial arts, and indoor climbing) could promote specific changes in cognitive functions [125]. Practising specific sports could lead to differential benefits on cognitive processing. Therefore, selecting the most appropriate PA depending on individual demands would be helpful.

fMRI represents the best available neuroimaging method in neurological fields. However, some studies preferred resting-state fMRI in subjects unable to perform tasks accurately because of physical or cognitive impairment, eliminating the confounding effects due to differences in task performance [153,154]. Children showed resting-state networks more defined by anatomical proximity, whereas adults showed patterns more related to functional relationships [155]. However, fMRI reproducibility can be compromised by experimental factors, e.g., length of scan, cognitive task design, and motion artefacts. In addition, total sleep duration and sleepiness could affect the measures of the cortical hemodynamic response; cognitive performance is linked to some biopsychosocial factors, such as circadian rhythms [156,157,158], tiredness [159], and level of arousal.

The current review was subjected to the following limitations. We did not include behavioural mechanisms since only some studies dealt with this aspect. Exercise-induced positive benefits on sleep and mood could enhance cognitive function [160]. In addition, motivation, fatigue, and perception of pain could affect training compliance and, thus, brain health [161]. Individuals with serious psychological disorders, with a potential lack of motivation were usually excluded from the studies. Future studies should investigate how behavioural mechanisms could regulate the effects of PA on cognition. Our research was conducted in a single database. Therefore, it could be possible that potentially eligible papers were omitted.

Overall, the results tended to need more generalisability; brain changes did not manifest equally or uniformly throughout the brain, probably because the studies differed in terms of their study samples, imaging methods, and type of exercises. Several study limitations should be noted. The fMRI studies generally encompassed a small sample size with a homogeneous population. Moreover, including participants with higher educational levels could be a confounding variable. Most fMRI studies limited their sample to right-handed people and did not investigate the potential different brain activations between right- and left-handed individuals. Few papers dealt with the effects of chronic physical exercise in adults [161]. Moreover, these studies included a large range of ages, from young adults to middle-aged people, without considering the hormonal changes that occur in the passage from young to middle-aged adulthood [161,162]. The duration of the studies was limited to a maximum of one year. Hence, assessing whether the PA-induced results remain stable over time following PA cessation would be interesting.

Given the differences in study design, cognitive tests, neuroimaging methods, and study samples in the reviewed studies, future studies should be drawn to overcome these limitations and to better understand the mechanisms underpinning the impact of PA on cognition. **More well-designed randomised controlled trials with appropriate sample sizes and post-PA follow-up assessments should be conducted to increase the findings’ reliability.**

However, despite the heterogeneity among the research, **our findings provide a further and current understanding of PA-induced mechanisms** and highlight the strength of exercise-related effects on both the brain and cognition across all age groups. PA may be considered a powerful tool to preserve and foster neurocognitive functioning throughout a person’s lifetime. **Furthermore, our review supports the importance of practising a specific type of PA in selected people, for instance, in patients with psychological disorders, to gain better cognitive performance.**

Regular PA could be an essential and decisive protective factor against cognitive decline and dementia in the elderly. It should be recommended during childhood and adolescence, characterised by rapid growth and development. **Thus, public health initiatives should be implemented to raise awareness among people of different ages regarding the impact of PA on mental health.**

## 5. Conclusions

Regular PA leads to positive effects in multiple cognitive domains at every stage of life, and neurological tests and neuroimaging widely support this evidence. A healthy lifestyle and proper PA reduce inflammatory states; increase the presence of synaptogenic, angiogenic, and neurotrophic factors; and improve cognitive functions (e.g., memory and executive function) and functional connectivity. It can be suggested that a particular exercise type could provide better cognitive improvements for a selected target of subjects, for instance, light-intensity exercise in patients with depression or neural diseases. However, our findings are inconclusive because of the heterogeneity among the papers reviewed here. Future well-designed studies should select the most suitable type of PA for every age group and to detail the impact of PA in adults. Therefore, correct and constant PA is fundamental for adolescents’ and children’s physical, psychophysical, and mental well-being and should be promoted and implemented in educational and recreational places. In addition, regular PA may prevent the onset of diseases related to cognitive decline with age.

## Figures and Tables

**Table 1:** Summary of studies investigating acute exercise.

Study	Sample	Physical Activity	Methods	Main Findings
Coelho et al., 2014 [40]	21 AD * patients (76.3 ± 6.2 yrs) 18 healthy controls (74.6 ± 4.7 yrs)	Treadmill	Treadmill grade, time to exhaustion, VO2, maximal lactate, Baecke Questionnaire	Increase in BDNF * plasma levels
Won et al., 2019 [41]	26 healthy older adults (65.9 ± 7.2 yrs)	30 min of cycling	fMRI * scan	Activation of semantic memory
Won et al., 2019 [42]	32 healthy older adults (66.2 ± 7.3 yrs)	30 min cycling	fMRI scan	Activation in the left inferior frontal gyrus and inferior parietal lobule
Voss et al., 2020 [43]	34 healthy older adults (67.1 ± 4.3 yrs)	20 min of light and moderate cycling	fMRI scan	Improvements in hippocampal–cortical connections and working memory
Suwabe et al., 2018 [44]	36 healthy young adults (20.9 ± 1.8 yrs)	10 min of light exercise	fMRI scan	Increase in connectivity between dentate gyrus and cortical regions
Li et al., 2014 [45]	15 female students (19–22 yrs)	20 min of moderate exercise	fMRI scan	Activation in the right middle prefrontal gyrus, right lingual gyrus, and left fusiform gyrus; deactivations in anterior cingulate cortex: left inferior frontal gyrus and right paracentral lobule
Marian Bosch et al., 2020 [46]	15 healthy male young adults (23.7 ± 4.02 yrs)	30 min of moderate exercise/15 min of vigorous exercise	fMRI scan	Significant improvements in motor sequence memory (high intensity)/improvements tending towards significance (moderate intensity)
Perini et al., 2016 [47]	84 healthy male young adults: 44 (23.0 ± 2.2 yrs) in the orientation discrimination task group and 40 (22.9 ± 2.2 yrs) in the motor task group	30 min of exercise	Physiological and behavioural tests	Gradual up-regulation of a functional network
Mehren et al., 2019 [48]	20 ADHD * patients (29.9 ± 9.5 yrs) 20 healthy controls (29.0 ± 7.4 yrs)	30 min of moderate cycling	fMRI scan	Improvements in reaction times and attention in ADHD patients; activation in frontal and sensorimotor regions in patients and controls
Mehren et al., 2019 [49]	64 healthy young adults: 32 participants (29.3 ± 8.5 yrs) in the moderate-intensity group; 32 participants (28.6 ± 7.7 yrs) in the high-intensity group	Moderate- and high-intensity cycling	fMRI scan	Activation in the insula, superior frontal gyrus, precentral gyrus, and supplementary motor area in the moderate-intensity group
Schmitt et al., 2019 [50]	21 male athletes (27.2 ± 4.2 yrs)	30 min of low- and high-intensity exercise	fMRI scan	Reduced activation in posterior cingulate cortex/precuneus for low intensity; reduced activation in the caudate nucleus and ventral anterior putamen for high intensity
Li et al., 2019 [51]	12 healthy high-fit and 12 healthy low-fit female students	30 min of aerobic exercise	fMRI scan	Activation in the right cerebellum and subcortical regions
Choi et al., 2016 [52]	37 ADHD children; 18 healthy controls (12.8 ± 0.79 yrs)	13 min of aerobic stretching and moderate exercise	EEG *	Increased alpha activity; decreased theta activity
Chen et al., 2016 [53]	9 healthy children (10 yrs)	30 min of moderate cycling	fMRI scan	Activation in bilateral parietal cortex: left hippocampus and bilateral cerebellum
Chen et al., 2017 [54]	9 healthy children (10 yrs)	30 min of moderate cycling	fMRI scan	Connectivity between left cerebellum and right inferior frontal gyrus
Metcalfe et al., 2016 [55]	30 BD * adolescents (16.8 ± 1.4 yrs); 20 healthy controls (16.1 ± 1.5 yrs)	20 min of recumbent cycling	fMRI scan	Deactivation in the left inferior frontal gyrus, right frontal pole, temporal pole, hippocampus, and right amygdala

* AD: Alzheimer’s disease; ADHD: attention deficit hyperactivity disorder; BD: bipolar disorder; BDNF: brain-derived neurotrophic factor; EEG: electroencephalography; fMRI: functional magnetic resonance imaging.

**Table 2:** Summary of studies investigating chronic exercise among older adults.

Study	Sample	Physical Activity	Methods	Main Findings
Engeroff et al., 2018 [73]	Healthy older adults (>65 yrs)	Moderate-to-vigorous exercise	MRS *-based marked	Increase in BDNF * and hippocampus volume
Wheeler et al., 2020 [74]	67 healthy older adults (67 ± 7 yrs)	6 days of moderate exercise	Cognitive testing	Increase in BDNF, working memory, and executive function
Gaitán et al., 2021 [75]	23 late middle-aged adults (mean age 65 yrs)	26 weeks of treadmill training	Cognitive function test and Enzyme-Linked Immunosorbent Assay (ELISA)	Increased plasma Cathepsin B; unchanged serum klotho
Neale et al., 2017 [76]	95 healthy older adults (65–92 yrs)	Light exercise	EEG *	Association between neural signature and environment
Gogniat et al., 2022 [77]	47 healthy older adults (>65 yrs)	7 days of exercise	Neuropsychological tests and fMRI * scan	Increased DMN * and DAN * functional connectivity
Gogniat et al., 2022 [78]	51 healthy older adults (>65 yrs)	Light exercise	Neuropsychological tests and fMRI scan	Relationship between low DMN/DAN anti-correlations levels and better executive function
Pieramico et al., 2012 [79]	30 healthy older adults (60–75 yrs): 15 participants; 15 controls	6 months of structured multimodal activities (cognitive, aerobic, and sensorial stimuli and fun recreational activities)	fMRI scan	Improvements in cognitive performance and reorganization of functional connectivity
Li et al., 2014 [80]	34 healthy male older adults: 17 (68.6 ± 5.7 yrs) participants; 17 (71.7 ± 4.0) controls	6 weeks of multimodal activities (Tai Chi and counselling group); lecture for the control group	Cognitive tests and fMRI scan	Improved functional connectivity between the medial prefrontal cortex and medial temporal lobe
Tao et al., 2016 [81]	62 older adults: 21 in the Tai Chin Chaun group; 16 in the Baduanjin group; 25 in the control group	12 weeks of Tai Chin Chuan or Baduanjin exercise	Memory function measurement and fMRI scan	Increased memory quotient; improved functional connectivity between the hippocampus and medial prefrontal cortex
Dorsman et al., 2020 [82]	212 older adults (73.3 ± 6.2 yrs)	7 days of exercise	fMRI scan	Greater frontal-subcortical and within-subcortical network synchrony
Ji et al., 2017 [83]	24 older adults: 12 (67.0 ± 6.40 yrs) participants; 12 (73.0 ± 8.0 yrs) controls	6 weeks of exercise	Cognitive tests, and MRI * and fMRI scans	Improved memory and executive function; increased posterior cingulate volume; higher connectivity between the striatum and cingulate, temporal, parietal, and occipital regions
Voss et al., 2010 [84]	65 older adults: 30 (67.3 ± 5.8 yrs) participants; 35 (65.4 ± 5.2 yrs) controls	6 and 12 months of moderate aerobic exercise	Cognitive tests and fMRI scan	Improved executive function; increased functional connectivity within DMN and FEN *
Flodin et al., 2017 [85]	47 older adults: 22 (68.4 ± 2.6 yrs) participants; 25 (69.16 ± 3.0 yrs) controls	6 months of aerobic exercise	fMRI scan	Decreased connectivity between left hippocampus and contralateral precentral gyrus; Better connectivity between right mid-temporal areas and frontal and parietal region
Voss et al., 2019 [86]	189 healthy older adults (65.4 ± 4.4 yrs)	6 months of aerobic exercise (dance and walk)	fMRI scan	Increased salience network connectivity via nutritional supplementation
Kimura et al., 2013 [87]	72 healthy older adults (70.3 ± 4.0)	3 months of short and long brisk walking	fMRI scan	Activation in left prefrontal and parietal regions and in dorsolateral prefrontal cortex
Bugg et al., 2011 [88]	52 healthy older adults (69.0 ± 6.7 yrs)	exercise (running or walking) practiced over the last 10 yrs	MRI scan	Larger frontal volume and medial temporal lobule
Papenberg et al., 2016 [89]	414 healthy older adults	exercise practiced in the last 12 months	MRI scan	Correlation between physical inactivity and small grey matter volume
Well et al., 2013 [90]	14 MCI patients: 9 participants; 5 controls	8 weeks of mindfulness	fMRI scan	Increased connectivity between posterior cingulate cortex, bilateral medial prefrontal cortex, and left hippocampus
Eyre et al., 2016 [91]	25 MCI * patients: 14 in the yoga group; 11 in the memory enhancement training group	12 weeks of yoga	fMRI scan	Greater connectivity between the DMN and medial frontal cortex, pregenual anterior cingulate cortex, right middle frontal cortex, posterior cingulate cortex, and left lateral occipital cortex
Tao et al., 2019 [92]	47 MCI patients: 20 (66.17 ± 4.17 yrs) in the Baduanjin group; 17 (64.32 ± 2.60 yrs) in the brisk walking group; 20 (65.97 ± 5.66 yrs) in the control group	24 weeks of exercise (Baduanjin and brisk walking)	Montreal cognitive test, and MRI and fMRI scans	Improved cognitive function; greater hippocampus grey matter volume; higher connectivity between the hippocampus and right angular gyrus
Suo et al., 2016 [93]	100 MCI older adults (70.1 ± 6.7 yrs)	6 months of resistance training	Neuropsychological tests, and MRI and fMRI scans	Better global cognition; greater cortical thickness in the posterior cingulate; improved connectivity between the hippocampus and superior frontal cortex
Hsu et al., 2017 [94]	21 SIVCI * older adults	6 months of aerobic exercise	fMRI scan	FPN * linked to better mobility performance
Veldsman et al., 2017 [95]	62 stroke patients (67 ± 12.6 yrs) 27 healthy controls (68.0 ± 5.94 yrs)	3 months of exercise	fMRI scan	Increased connectivity of superior parietal lobule in DAN

* BDNF: brain-derived neurotrophic factor; DMN: default mode network; DAN: dorsal attention network; EEG: electroencephalography; FEN: frontal executive network; FPN: frontoparietal network; fMRI: functional magnetic resonance imaging; MCI: mild cognitive impairment; MRI: magnetic resonance imaging; MRS: magnetic resonance spectroscopy; SIVCI: subcortical ischemic vascular cognitive impairment.

**Table 3:** Summary of studies investigating chronic exercise among adults.

Study	Sample	Physical Activity	Methods	Main Findings
Goldin et al., 2012 [105]	42 adults with SAD * (32.88 ± 7.97 yrs): 24 in the MBSR group; 18 in the aerobic group	8 sessions of weekly MBSR; 8 weeks of aerobic exercise	fMRI * scan	Greater brain responses in the posterior cingulate cortex in the MBSR group
Goldin et al., 2013 [106]	42 adults with SAD (32.88 ± 7.97 yrs): 24 in the MBSR * group; 18 in the aerobic group	8 sessions of weekly MBSR; 8 weeks of aerobic exercise	fMRI scan	Reduced negative emotions; increase in attention-related parietal cortical regions in the MBSR group
Gourgouvelis et al., 2017 [107]	16 adults: 8 patients with depression and anxiety (37.25 ± 8.0 yrs); 8 healthy controls (20.63 ± 1.19 yrs)	8 weeks of moderate intervention: resistance training, and mild to vigorous aerobic session	fMRI scan	Reduced hippocampal activity in patients
Huang et al., 2021 [108]	70 adults (18–50 yrs): 38 StD patients; 32 healthy controls	8 weeks of moderate aerobic exercise	fMRI scan	Reduced right inferior parietal lobule activity in StD * patients
Stern et al., 2019 [109]	132 healthy adults (20–67 yrs)	6 months of aerobic exercise/stretching and toning	MRI scan	Increased cortical thickness in the left caudal middle frontal cortex Brodmann area in the aerobic group
Bashir et., 2021 [110]	45 healthy adults (19–27 yrs): 25 in the exercise group; 20 in the control group	6 months of aerobic and anaerobic exercise	MRI scan	Increased cortical thickness in left peri calcarine area, left superior parietal area, right rostral middle frontal, and right lateral occipital gyrus
Kaiser et al., 2022 [111]	45 healthy adults (18–30 yrs)	12 weeks of high- vs. low-intensity exercise	MRI scan	Increased left hippocampal and decreased right hippocampal volume after vigorous exercise
Fontes et al., 2013 [112]	7 healthy male adults (26.6 ± 4.0 yrs)	6 sessions of cycling	fMRI scan	Relation between posterior cingulate cortex and precuneus and higher levels of perceived exertion
Ishihara et al., 2020 [113]	1033 healthy adults (22–37 yrs)	Not specified	fMRI scan	Increased functional connectivity within DMN and FPN *
Nakagawa et al., 2020 [114]	58 healthy adults (22.4 ± 2.4)	Moderate-to-vigorous exercise vs. low-to-moderate exercise	Cognitive tests	Better cognitive performance in moderate-to-vigorous group
Bezzola et al., 2012 [115]	32 healthy middle-aged adults (51.2 ± 7.2 yrs): 11 in the golf group; 11 in the control group	40 h of golf training	fMRI scan	Reduction in neuronal recruitment in the right and left dorsal premotor cortex
Wadden et al., 2013 [116]	10 healthy middle-aged adults (64.7 ± 8.5 yrs)	7 days of exercise	fMRI scan	Bilateral cerebellar activation
Pensel et al., 2018 [117]	25 healthy middle-aged adults (52.21 ± 6.39 yrs)	6 months of aerobic exercise	fMRI scan	Bilateral frontal activation

* DMN: default mode network; fMRI: functional magnetic resonance imaging; FPN: frontoparietal network; MRI: magnetic resonance imaging; MBSR: mindfulness-based stress reduction; SAD: social anxiety disorder; StD: subthreshold depression.

**Table 4:** Summary of studies investigating chronic exercise among adolescents and children.

Study	Sample	Physical Activity	Methods	Main Findings
Kjellenberg et al., 2022 [123]	1139 adolescent (13.4 ± 0.3 yrs)	7 days of moderate-to-vigorous exercise	Cognitive tests	Better cognitive function
Kamijo et al., 2011 [124]	43 children (7–9 yrs): 22 in the exercise group; 21 in the control group	9 months of aerobic exercise	Cognitive tests	Improved working memory
Quinzi et al., 2022 [125]	64 children	Racket sport, martial arts, and indoor climbing	Cognitive tests	Improved specific domains related to exercise type
Hillmann et al., 2014 [126]	221 children (7–9 yrs): 109 in the exercise group; 112 in the control group	9 months of aerobic exercise	fMRI * scan	Improved executive control and brain activity
Chaddock-Heyman et al., 2013 [127]	23 children (8.9 ± 5.8)	9 months of moderate-to-vigorous aerobic exercise	fMRI scan	Reduced activation in the right anterior prefrontal cortex
Chaddock-Heyman et al., 2013 [128]	143 children (8.7 ± 0.55)	9 months of aerobic exercise	MRI * scan	Improved white matter microstructure in the genu of the anterior corpus callosum
Logan et al., 2021 [129]	206 children (8–10 yrs): 103 normal weight; 103 obese	9 months of aerobic exercise	EEG *	Reduced neuroleptic indices in obese
Ortega et al., 2022 [130]	90 overweight children (8–10 yrs)	20 weeks of high-intensity aerobic exercise	Standardised tests and MRI scan	Improved intelligence and cognitive flexibility; unidentified structural changes
Davis et al., 2011 [121]	19 overweight children (9.8 ± 1.0 yrs)	3 months of regular aerobic exercise	fMRI scan	Increased bilateral prefrontal cortex activity. Reduced bilateral posterior parietal cortex activity
Kraff et al., 2014 [131]	43 overweight children (9.8 ± 0.8 yrs)	8 months of aerobic exercise	fMRI scan	Reduced activation in prefrontal and parietal areas; increased activation in the frontal gyrus and anterior cingulate
Kraff et al., 2014 [132]	22 overweight children (9.5 ± 0.7 yrs): 13 in the exercise group; 9 in the control group	8 months of aerobic exercise	fMRI scan	Reduced synchrony in motor, default mode, and cognitive control networks; increased synchrony only between the motor network and frontal regions
Kraff et al., 2014 [133]	18 overweight children (9.7 ± 0.7 yrs): 10 in the exercise group; 8 in the control group	8 months of aerobic exercise	DTI *	Increased white matter integrity in the bilateral superior longitudinal fasciculus
Schaeffer et al., 2014 [134]	18 overweight children (9.7 ± 0.7 yrs): 10 in the exercise group; 8 in the control group	8 months of aerobic exercise	DTI	Positive change in bilateral uncinate fasciculus

* DTI: diffusion tensor imaging; EEG: electroencephalography; fMRI: functional magnetic resonance imaging; MRI: magnetic resonance imaging.

## Data Availability

Not applicable.
